# Caregivers’ experience of sleep management in Smith–Magenis syndrome: a mixed-methods study

**DOI:** 10.1186/s13023-021-02159-8

**Published:** 2022-02-04

**Authors:** Georgie Agar, Stacey Bissell, Lucy Wilde, Nigel Over, Caitlin Williams, Caroline Richards, Chris Oliver

**Affiliations:** 1 School of Psychology, University of Birmingham, 52 Pritchatts Road Edgbaston, Birmingham, B15 2TT UK; 2 Cerebra Network for Neurodevelopmental Disorders, Birmingham, UK; 3 The Open University, Milton Keynes, UK; 4 The Smith-Magenis Syndrome (SMS) Foundation UK, Livingston, UK; 5 Centre for Educational Development Appraisal and Research, University of Warwick, Coventry, UK

**Keywords:** Caregivers, Parents, Smith–Magenis syndrome, Sleep, Safety, Qualitative, Disability

## Abstract

**Background:**

Smith–Magenis syndrome (SMS) is a rare genetic syndrome associated with a unique profile of early morning waking and daytime sleepiness. Children with SMS evidence high rates of self-injury and aggression and have a preference for adult over peer attention, with strong motivation to interact with a particular caregiver. In addition, people with SMS have lower adaptive functioning skills relative to cognitive abilities and demonstrate high levels of impulsivity. Taken together, these factors may result in individuals being awake overnight requiring vigilant caregiver supervision. Despite these complexities, no study has described the strategies caregivers take to keep their children with SMS safe overnight or considered the impact of these experiences on caregivers or the wider family.

**Methods:**

The current study used a mixed-methods approach to consider sleep management strategies and challenges for caregivers of people with SMS at different ages. Caregivers completed an international online survey about sleep management and related difficulties, use of interventions and access to services and support. Semi-structured interviews were conducted with 14 caregivers in the UK to increase understanding of caregiver experiences and priorities for change in the UK context. Interviews were transcribed verbatim and coded using thematic analysis.

**Results:**

Evidence from the online survey (n = 40) revealed wide-ranging impacts of poor sleep on the person with SMS and the wider family. Only 5% of caregivers reported that the sleep problems had no impact on their child, and 76% reported a moderately or extremely significant impact on themselves. For some individual caregivers, sleep management difficulties improved over time whereas for others no change was reported. Weekly respite emerged as the ideal provision for 49% of caregivers, although only 14% had access to this. The majority of caregivers (54%) received no respite. Thematic analysis of qualitative interviews revealed interactions between aspects of the behavioural phenotype of SMS which may contribute to complex and unusual presentations in relation to sleep management and safety.

**Conclusions:**

Caregivers’ priorities for sleep management and support were delineated, with key implications for services in terms of the use of SMS-sensitive strategies and respite provision.

**Supplementary Information:**

The online version contains supplementary material available at 10.1186/s13023-021-02159-8.

## Introduction

Smith–Magenis syndrome (SMS) is a rare genetic syndrome which occurs in approximately 1 in 25,000 live births [1]. It is caused by deletion or variation to the retinoic acid induced 1 gene on chromosome 17p11.2 [2, 3] an area implicated in the regulation of several circadian genes [4]. SMS is associated with a well-defined behavioural phenotype which includes elevated rates of self-injurious and aggressive behaviour, impulsivity and preference for adult attention, often from a specific caregiver [5–9] Most people with SMS evidence mild to moderate intellectual disability [10] with relative weakness in adaptive functioning [11].

Perhaps the most striking feature of SMS is the profile of excessive daytime sleepiness and early morning waking [12–15] which is associated with the suggestion of an ‘inverted’ circadian rhythm in this group [16, 17]. This pattern is thought to be a result of dysregulation of the retinoic acid induced 1 gene [18]. This dysregulation is evident in the contrast between the timing of exogenous melatonin release of people with SMS compared to typically developing controls but lack of difference in the volume of synthesis [16]. In addition, sleep is objectively poorer in people with SMS than their age-matched typically developing peers [14, 15].

Taken together, these aspects of the phenotype may result in a person with SMS waking early in the morning with strong motivation to interact with a caregiver immediately, contributing to behaviours such as self-injury, aggression and temper outbursts. Concerns about impulsivity and adaptive functioning may increase the need for supervision, requiring caregivers to wake early which may be burdensome and result in less sleep than desired [19]. Indeed, sleep disturbance is the strongest predictor of challenging behaviour in people with SMS [20, 21] and has been associated with parent stress in SMS and other genetic syndromes with a similar prevalence of sleep disturbance such as Angelman syndrome [22, 23]. Several studies have begun to explore the wellbeing of caregivers of people with SMS [19, 24, 25] but none of these have explored directly the impact of managing sleep disturbance on caregivers of people with SMS. This is critical as the complex behavioural profile of SMS, which may not be suited to typical sleep management approaches, provides a unique set of challenges for people with SMS and their families relating to sleep safety and management.

Interestingly, Heald [26] found that mothers of children with SMS had higher perceived stress, and higher anxiety and depression than normative scores, but these variables were not directly associated with child or parent objective sleep parameters. Rather, wellbeing was significantly correlated with mothers’ perception of their own sleep disturbance. Therefore, it may be that factors which contribute to mothers’ perceived experience of sleep disruption, such as the unpredictability of sleep patterns or the broader experience of caregiving for their child overnight, have a more significant impact on parent wellbeing than the total amount of sleep. First-hand accounts of the challenges in caring for people with SMS can inform these interpretations and document the complexity of the atypically severe sleep management problem. Through qualitative analysis of caregiver experiences, interactions between different components of the behavioural phenotype and their summative effects can be explored, to generate models that might then be tested empirically.

To date, no studies have directly examined caregiver experiences and strategies for managing sleep in people with SMS, despite the complexity of the behavioural phenotype and marked profile of sleep disturbance in this group. Therefore, the aims of this study are:

1. To describe the experiences of caregivers of children and adults with SMS with regard to sleep management and safety at different ages.
2. To describe how interactions between aspects of the behavioural phenotype of SMS may contribute to complex and unusual presentations of strategies for sleep management and safety.
3. To delineate caregiver priorities for intervention and identify barriers to support, thus informing sleep management and safety policy for people with SMS.

## Methods

We used a mixed-methods approach to explore caregiver experiences of sleep safety and management. The study was approved by the University of Birmingham ethics board. Forty caregivers completed an online survey regarding their experiences of managing the sleep of their child with SMS, which was advertised online via the Smith–Magenis Syndrome (SMS) Foundation UK, the research team’s website and social media, and several international SMS research and family conferences. The survey was designed in collaboration with the Smith–Magenis Syndrome (SMS) Foundation UK, but the survey was open to any caregiver of an individual with SMS of any age, living in any country. Families from the UK, USA, Europe and Australia took part. Of the forty respondents, 27 were mothers of people with SMS and 7 were fathers. One mother and father completed the survey together, and four reported that they were a ‘parent’ of a person with SMS. Caregivers reported on 39 children and adults with SMS; three toddlers aged 0–3 years, nine young children aged 4–8 years, 11 older children aged 9–12 years, three adolescents aged 13–17 years and 13 adults aged ≥ 18 years.

In order to provide a broad picture of concerns around the impact of poor sleep and sleep management for caregivers of people with SMS, the authors developed an online survey (see Additional file 1). The survey comprised background questions relating to the number of people the respondent cared for, the living and sleeping arrangements of the person with SMS and 11 Likert scale questions which considered the severity of poor sleep at each stage of their life. The survey also asked about the impact of poor sleep on the person with SMS, the caregiver and the wider family. Caregivers were also asked to use free-text boxes to share their experiences of how managing sleep had changed over time, including anything that consistently improved or decreased their child’s sleep quality, strategies they used to manage their child’s sleep and keep them safe at night, and the worst impact of their child’s poor sleep on themselves and their child. Quotes from these free-text boxes are presented in italics throughout.

In addition, 10 face-to-face interviews with 14 caregivers were conducted in the UK, to increase understanding of caregiver experiences and priorities for change in the UK context. The majority of caregivers were recruited to the interview aspect of the study after completing the online survey and consenting to future parts of the study. Several caregivers were also recruited through opportunistic sampling at the Smith–Magenis Syndrome (SMS) Foundation UK family conference. All interviews were conducted by the co-first author (SB). Two of the interviews included parent dyads. In another, one parent and two siblings of an adult with SMS took part. The characteristics of these caregivers are reported in Table 1. Given the rarity of the syndrome, minimal information is provided and age information reflects ages advocated by McDonagh et al., [27]. Interviews were semi-structured and designed to give caregivers the opportunity to expand on some areas of the online survey. All interviews were recorded and transcribed verbatim, then analysed using NVivo 12 software. This software allows the user to code transcripts using thematic analysis, according to ‘nodes’ of interest, which can be pre-determined or developed during analysis. In this study, the initial nodes were drawn from the key themes that emerged in the online survey: sleep at different ages, change over time, impact on the person with SMS, impact on the caregiver, impact on the wider family, safety concerns and strategies and other management strategies. In addition, the theme of ‘respite’ was added during analysis. Example of quotes from specific caregivers are used to illustrate the findings.

**Table 1:** Characteristics of caregivers who completed face-to-face interviews

Caregiver 1	Mother of an adolescent male with SMS
Caregiver 2	Father of an adolescent male with SMS
Caregiver 3	Mother of an adolescent female with SMS
Caregiver 4	Father of an adolescent female with SMS
Caregiver 5	Mother of an adolescent male with SMS
Caregiver 6	Mother of a female child with SMS
Caregiver 7	Mother of a female child with SMS
Caregiver 8	Mother of an adult male with SMS
Caregiver 9	Father of an adolescent female with SMS
Caregiver 10	Mother of an adolescent male with SMS
Caregiver 11	Father of an adolescent male with SMS
Caregiver 12	Mother of an adult female with SMS
Caregiver 13	Sister of an adult female with SMS
Caregiver 14	Sister of an adult female with SMS

## Results

### Sleep at different ages

**Fig. 1:**
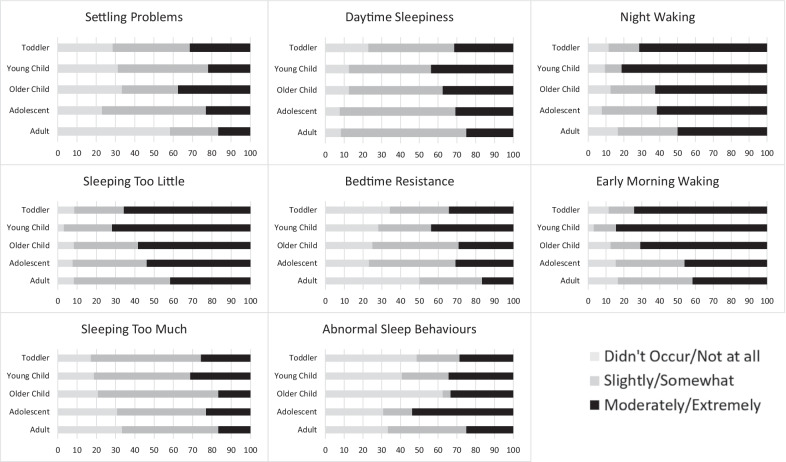
The percentage of caregivers experiencing difficulty managing each aspect of poor sleep at each age

The difficulty of managing each aspect of poor sleep at each age reported in the online survey is shown in Fig. 1.

The results in Fig. 1 demonstrate that of the 35 responses relating to sleep in individuals with SMS as toddlers, 23 (66%) caregivers found it moderately/extremely difficult to manage their toddler sleeping too little, 25 (72%) found it moderately/extremely difficult to manage their toddler’s wakings and 26 (74%) found managing their toddler’s early waking moderately/extremely difficult. In the interviews, several caregivers described issues with their child’s sleep starting from infancy, with the wakings difficult to manage at even a young age.

“Because everyone says a baby sleeps, a baby sleeps lots. [He] never slept lots as a baby, at all. The only time he’d sleep, is if I was walking him in a pram, and he might sleep for 2 or 3 hours in the night time, and that was it… [As a toddler] he used to wake up at 3 and 4, take his nappy off and throw it around the room, and wouldn’t come and wake anybody up, just cause hairy carey.” [Caregiver 1]

Of the 32 responses relating to sleep in young children with SMS, 23 (72%) caregivers found it moderately/extremely difficult to manage their young child sleeping too little. Twenty-six (81%) found it moderately/extremely difficult to manage their young child’s wakings and 27 (84%) found it moderately/extremely difficult to manage their young child’s early waking. Several families indicated that this was the most difficult age period for sleep.

“Between the age of 3 and 7, she was probably at her most challenging, and those were the hardest times, because she just couldn’t be left unsupervised through the night so we would have to be in there.” [Caregiver 3]

Of the 24 responses relating to older children, 14 (58%) caregivers found it moderately/extremely difficult to manage their older child sleeping too little. Fifteen (63%) found it moderately/extremely difficult to manage their older child’s wakings and 17 (71%) found it moderately/extremely difficult to manage their older child’s early waking. Some caregivers described that though their child still woke regularly at this age, they were more independent and their waking behaviour was easier to manage.

“She would still waken up but she decided that she would go back to her own bed [rather] than sleep in the parental bed, just stopped.” [Caregiver 9]

“By the time she started secondary school but her sleeping was improving then, she wasn’t coming into our room or your room particularly.” [Caregiver 12]

Of the 13 responses relating to sleep in adolescents with SMS, 7 (54%) caregivers found it moderately/extremely difficult to manage their adolescent sleeping too little and 8 (62%) found it moderately/extremely difficult to manage their adolescents’ waking. Six (46%) caregivers found it moderately/extremely difficult to manage their adolescents’ early waking and 7 (54%) found it moderately/extremely difficult to manage abnormal sleep behaviours. Of the 12 caregivers reporting on their experiences of managing their adult child with SMS’s sleep, 5 (42%) found it moderately/extremely difficult to manage their adult child sleeping too little. Six (50%) found it moderately/extremely difficult to manage their night waking, and 5 (42%) their early waking.

### Change over time

**Fig. 2:**
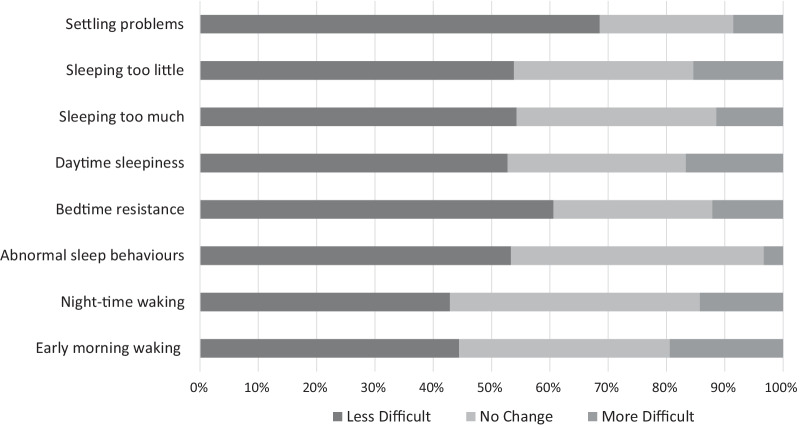
The percentage of caregivers who found each aspect of poor sleep more difficult, less difficult or the same level of difficulty over time

Figure 2 shows the extent to which caregivers felt these sleep difficulties became more or less difficult to manage over time.

Overall, data from the online survey revealed some evidence of improvements over time. For example, several caregivers reported that the worst period for managing sleep was when children were young (aged 3–7 years).

“It’s always that age band that seems to be the most tricky, and yes they come out of it a bit easier as they get older and mature, and slightly more independent.” [Caregiver 3]

Others reported the change was more gradual.

“She’s less demanding […] when she wakens up, so although it’s been a gradual change from one stage to the next, looking back, things are definitely better now at night than they were, say 10 years ago.” [Caregiver 9]

“So yeah, during the 16 years, 15 years, sleep has changed, now we’re getting to the point where some nights he’ll be alright and some nights he’s not you know.” [Caregiver 10]

However, a substantial number of caregivers felt that their child’s sleep had not changed at all over time. In one survey response, the caregiver stated “*I have accepted that my son’s sleep pattern won’t change…..I have adapted…so wake early and go to bed early*”. This was echoed in several of the face-to-face interviews, when asked how their child’s sleep had changed over time, caregivers responded:

“It’s still much and muchness hasn’t really changed at all.” [Caregiver 11]

“Probably not really, basically.” [Caregiver 8]

### Impact of sleep on the person with SMS

The impact of these sleep difficulties on the person with SMS was reported to be extremely difficult for 13 of the 37 respondents (35%), and moderately difficult for a further 10 respondents (27%). Only two (5%) caregivers reported that the sleep problems had no impact on their child. When asked to provide examples of the impact on their child in the survey, parents reported a range of incidents relating to safety of their child overnight including one child who had “*climbed out of a first floor bedroom window*” and one who “*nearly died of hypothermia…because she left the room in a hotel…by the time we found her she was unresponsive with a temperature in the 80s Fahrenheit*”.

**Fig. 3:**
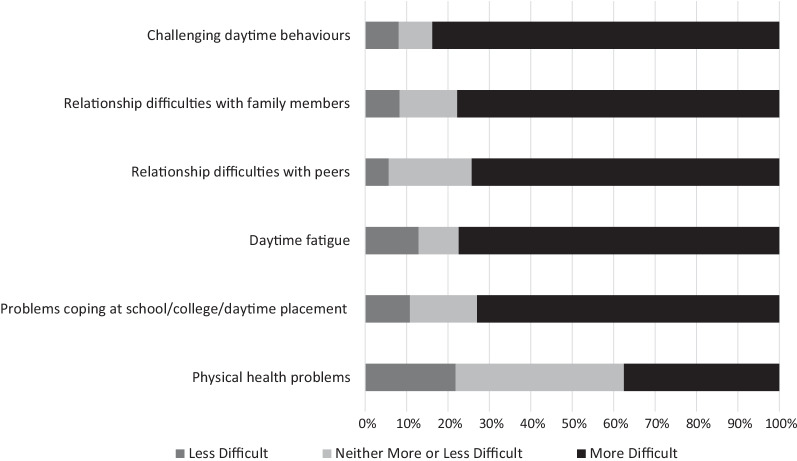
The percentage of caregivers who found each aspect of the person with SMS’ behaviour more or less difficult to manage as a result of their poor sleep

Figure 3 depicts the extent to which various behaviours became more or less difficult to manage in people with SMS as a result of their sleep difficulties.

Figure 3 demonstrates that the most difficult behaviour for caregivers to manage was challenging behaviour, with qualitative descriptions in the survey of “*terrible exhaustion at school with aggressive meltdowns*”, and severe self-injury overnight “*at its worst my daughter was up 102 times in the night, head banging the cot, led to bruising, throwing herself from one side of the cot to the other, screaming and hair pulling*”. The relationship between sleep and daytime challenging behaviour was also frequently described in several caregiver interviews, though this was not always a direct association.

“I think, it massively has an impact on her behaviour like the worse her sleep is the worse her behaviours are and I suppose as she gets older those behaviours become more destructive don’t they and more damaging.” [Caregiver 7]

“No there’s definitely, if she’s sleep deprived the night before, the behaviour the next day would be worse, doesn’t always correlate, she could have a perfect night’s sleep and still be badly behaved, so it wasn’t the only trigger.” [Caregiver 9]

In all the interviews, caregivers described the daytime fatigue their child with SMS experienced, including regularly falling asleep in the car and at mealtimes. For some individuals, this daytime fatigue seemed to escalate challenging behaviour.

“She very often will fall asleep in her dinner at lunch time and I mean literally like fall […] will literally just fall asleep.” [Caregiver 7]

“When he’s fatigued and angry he wants to lash out and his lashing out will be to annoy you in some way, so he’ll pick something that you’re really sensitive about.” [Caregiver 11]

### Impact of sleep on caregiver

**Fig. 4:**
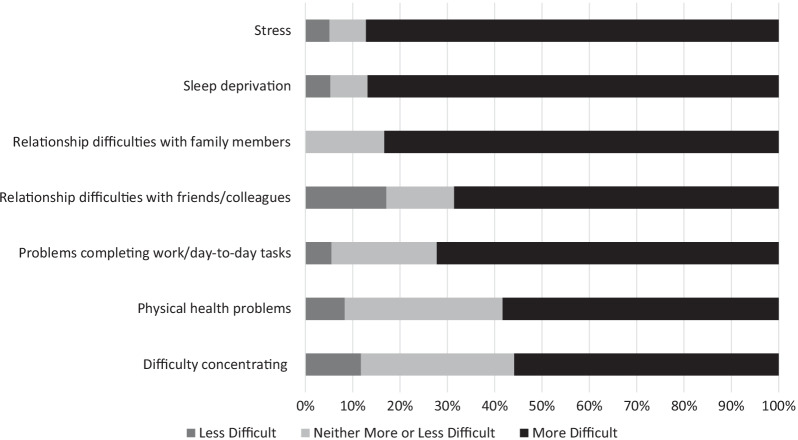
The percentage of caregivers who found each aspect of their own wellbeing more or less difficult to manage as a result of the person with SMS’ poor sleep

The majority of caregivers (29/38, 76%) reported that their child’s sleep difficulties also had an extremely significant (19/38, 50%) or moderate impact (10/38, 26%) on themselves. One caregiver explained: “*Chronic lack of sleep is hard to deal with on many levels. With a newborn, you know it's going to end someday. With SMS, there is no end in sight. Long term, pervasive lack of sleep affects my entire life—my ability to focus and get things done at work, my relationship with my husband and kids, relationships with other family and friends. It affects everything in my life and makes everything harder*.” Only two caregivers reported their child’s sleep difficulties had no impact on them as caregivers. Figure 4 demonstrates the impact of the person with SMS’ poor sleep on a range of caregiver outcomes.

The majority of caregivers experienced sleep deprivation and stress as extremely difficult to manage. In free text response, one caregiver explained, “*Exhaustion and fatigue have a direct impact on my job, some days I have severe 'brain fog'. Over the years I have suffered depression and anxiety for which I am now medicated. It just makes functioning on a daily basis so much more challenging.*”

In the face-to-face interviews, the impact on caregivers was explored further, with many caregivers describing their own sleep deprivation and how this impacted on their ability to function at work and support their child.

“Well that’s even worse, every hour of the day to be that strong and that’s the challenge for all of us, especially when you’re sleep deprived because you’re getting up really early and dealing with their aggressive behaviour and trying desperately to manage all these other things so that you can try and make them a bit more independent.” [Caregiver 5]

“Yeah you’re just on your knees with like tiredness but you’re still trying to look after the other two, still trying to go to work and it just yeah just…and it affects you doesn’t it because your like health massively like goes downhill as well…I worry about myself as well because like how much longer can you go on surviving when you barely like some days are fine and other days are really not but…Probably two hours sleep and that when you’ve got to do a day’s work as well.” [Caregiver 7]

“I was saying I’ve got to look after myself because I’m not even going to be here to fight his battles if I carry on not having that, no sleep, you know four hours a night is not going to make me a healthy person.” [Caregiver 8]

Caregivers also reported that their child’s sleep affected their stress levels and mental health.

“It’s the pressure of coping when you are aware that there isn’t a light at the end of a tunnel that this is going to go on and on and on and if you have the flu […] or if you're injured, you ain't got a choice you gotta carry on and that’s where it starts hurting when you have a bad day and you’re naturally fatigued and then… your behaviour starts then escalating and cascading into the marriage into your other children and you get into a vicious cycle.” [Caregiver 11]

“I was having major suicidal tendencies, you know, thoughts in my head because he was being illegally excluded and I had nowhere to go, and they battered us away.” [Caregiver 1]

“Basically I was so stressed out […] learning how to meditate, destress, everything, that takes the pressure off. It's kind of the only way I've done it […] that was a change basically because I was on antidepressants and all the rest.” [Caregiver 8]

Some caregivers had also experienced difficulties in their relationships with their partner, family members and friends as a result of their child’s poor sleep.

“And a lot of our relatives don’t really understand either. They’re like ‘oh just keep her up longer and she’ll sleep’, no, she won’t…” [Caregiver 6]

“It broke my marriage up her sleep was that bad I think because [he] just couldn’t cope.” [Caregiver 7]

“People shun us mostly.” [Caregiver 11]

### Impact of sleep on wider family

**Fig. 5:**
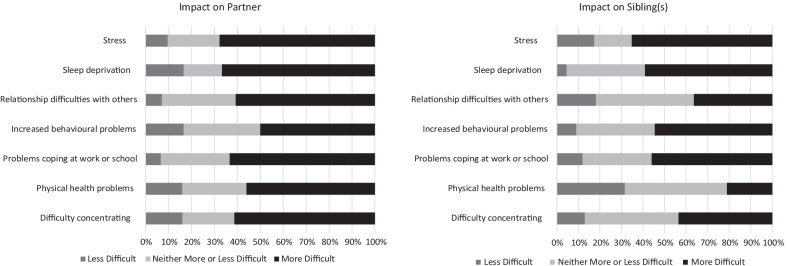
The difficulty of managing wellbeing for members of the wider family as a result of the person with SMS’ poor sleep

In Fig. 5, the impact of managing the sleep of the person with SMS on the wider family (partners and siblings) is reported. As with the caregiver responding to the survey, the most difficult issues for partners and siblings to manage were sleep deprivation and stress.

These issues were further explored in the caregiver interviews, with some caregivers reporting that their other children experienced sleep deprivation as a result of their child with SMS’ poor sleep.

“Yeah, he’s tired in the mornings. There are mornings where he’s like ‘Mummy, [sibling with SMS] woke me up’ you can tell that if he’d been allowed to sleep he would have slept. So now he’s into the habit of getting up really early because it’s something that he’s always done.” [Caregiver 6]

“Because she used to be a lot more active at night, like she used to come into my room when I would live at home and she would wake me up a lot […] Like sometimes I would turn up in the morning like ‘you woke me up three times last night and came through at 3’.” [Caregiver 14, a sibling of an adult female with SMS]

However, some caregivers reported that siblings appear to have adapted to the person with SMS’ poor sleep.

“It was always disturbances in the night. I think he managed quite well to learn to sleep ignoring the noise that was going on basically. [Caregiver 8]

She’d be woken up by [child with SMS] and then but do you know what I think the body is a brilliant thing because she’ll sleep through anything now and I think her body has a switch.” [Caregiver 10]

Others suggested that parenting their child with SMS and managing their sleep may have impacted on their ability to care for their other children in the way they would like to. As one caregiver on the online survey reported, “*Life revolves around SMSer*”.

“She [sibling] doesn’t have sleep deprivation she has attention deprivation…we will still defer to [child with SMS] most of the time because we know a lot of the time [child with SMS] can’t control it and other times it’s just easier to manage [sibling’s] disappointment than [child with SMS’] anger.” [Caregiver 11]

### Safety concerns and strategies

In the online survey, caregivers described a range of concerns around the safety of their child with SMS, and various strategies to mitigate these concerns overnight (see Table 2).

**Table 2:** Safety concerns and strategies reported by caregivers in the online survey

	Number	Example
*Concerns*		
Trying to ‘cook’	6	“She tried to cook porridge in the microwave and set it to 99 min, the microwave caught fire!”
Self-injury	5	“She has head butt the floor in a rage and bitten her hands to the point she has drawn blood.” “Smashed a hole in the wall in her room with her head”
Foraging for food	4	“Or eating anything and everything out of the fridge or cupboards.”
Destruction of property	4	“He flushed toys down the toilet and flooded our home.”
Trying to leave the house over night	2	“She nearly died of hypothermia when she was little because she left the room in a hotel in New Hampshire and got locked between the fire door and outdoors. By the time we found her she was unresponsive with a temperature in the 80s Fahrenheit.”
Climbing up furniture/windows	2	“Climbed out of 1st floor bedroom window at 2 years.”
Fire starting	2	“Trying to set house on fire.”
Interfering with plugs/plumbing	1	“Has dismantled plug sockets and cut wires unscrewed plumbing with hot water.”
‘Helping’ with domestic chores	1	“She once decided to 'help' us with the ironing. She left the iron switched on and face down on the ironing board so that it burned a hole through it.”
Smearing	1	“Smearing following soiled nappy.”
Accessing sharp objects/matches	1	“Managing to get hold of matches and hurting her self. Spent a week in a burns unit. Matches were hidden!”
Falling out of bed/cot	1	“He used to rock his cot to get out of that and he had fallen out of his cot.”
Aggression	1	“Gave me a bloody nose by head butting me as I was trying to calm her and get her to sleep.”
*Strategies*		
Lock/stair gate on bedroom door	12	“We have locked gates on her room, we have no choice!”
Adapted bedroom to remove furniture, sharp items etc	10	“Room was stripped back to essential items due to disruption and destruction.”
Enclosed bed	8	“Sleeps in a high sided padded profiling bed and has a safety sleeper or respite and holidays.”
Video/baby monitor in individual’s room	6	“CCTV in his room so that we can see what he is doing.”
Co-sleeping	6	“We lock him in our room and he sleeps with us.”
Locking doors to other rooms in the house	5	“Kitchen door is also locked at night.”
Constant supervision	5	“I always wake when he wakes.”
Locked windows	4	“Installed special front door and windows so he can't open them.”
Strategies to help child self-manage (e.g. use of iPad overnight)	3	“Once he has his iPad he is ok and will self manage in his room until 6:30.”
Hiding keys/food/potentially dangerous objects	3	“We have a 'lock down' routine before we go to bed every night. Always careful not to leave something out that could be potentially harmful to her, or endanger everyone else. It requires us to always be vigilant and careful.”
Alarms on doors	2	“We have doors to rooms with dangerous appliances/things like the kitchen alarmed.”

These concerns and strategies were further discussed in caregiver interviews, with many caregivers describing their concerns around their children’s behaviour and the need to monitor their child overnight to keep them safe. One caregiver described the need to “*sleep with one eye open*” in the online survey.

“When I realised he could get on the window ledge and open the windows, that was terrifying and that’s another reason why that works what we’ve done now, put the shutter on the outside, I know he can open the window and bang it but it only opens an inch so he cannot get out and get out there. Yeah and likewise when he was taking his bed apart, […] that’s a real safety issue so all of these things are safety issues.” [Caregiver 5]

“I’m actually really worried that she’s going to climb over the stairgate and she’ll be down the stairs and she’ll be in my knife drawer and all things like that […] We’ve got a video monitor on her as well so we can see what she’s doing at all times. We did try, you know you can get those alarm sensors that go under beds, we had one with our first child as well so you know if he stopped breathing in the night. We tried that with [child with SMS] but you know it was alarming every 5 minutes…”[Caregiver 6]

“If your child is safe at night time and you know they’re safe then as a parent you can sleep. If you’re worried about that child escaping then you’re not going to sleep and then you can’t get through the whole of the day can you, that’s how I look at it.” [Caregiver 10]

Caregivers often reported adapting their children’s bedrooms and access to other rooms in the house overnight in order to keep them safe.

“She’s not in a contained bed, and her room is not locked. So she has access to the house, but she does not have access to certain rooms in the house.” [Caregiver 3]

“So, certain things, at night time we have to then take out of the kitchen and lock in the garage. But, we’ve got used to actually separating our kitchen.” [Caregiver 1]

“In a normal house you don’t have a socket up at that height and the reason […] is so that we could put a clock in his room and plug it in without him wrapping the cable round his neck in the middle of the night because again that sort of thing is a real risk.” [Caregiver 5]

“We had a room built when he was 5 that has a window through from my room to his room, had all the sockets high up so he couldn’t reach them and everything was basically safe for him so he couldn’t get the windows and couldn’t get the door and all that so that was built up when he was 5 so we’ve pretty much, his room was kind of a safe space in itself.” [Caregiver 8]

Several caregivers reported that their children with SMS benefited from having an enclosed bed system, comprised of detachable fabric and meshing without any hard surfaces (also known as a ‘safe space’ or ‘safety sleeper’) where they could sleep at night.

“I think if he’s feeling safe, he feels better and that’s why probably he doesn’t sleep as well here as he does in his care home because he hasn’t got the safe space round him.” [Caregiver 8]

“The sleep tent will go with us when we’re not sleeping here and so that enables us to pop him in that and whilst it doesn’t stop him waking up and yelling for mummy to come to him at anything from 4 o’clock onwards, it does mean that I know that he’s safe in his room and not wandering about a strange person’s house.” [Caregiver 5]

“Get a safety sleeper literally like as soon as you can is my only like because there wasn’t, looking back there was nothing else that you could do because no matter how much reassurance you give her she doesn’t want it and she wasn’t able to sleep I don’t think was able to sleep for any longer than she was so at least they’ve got somewhere safe to be I think and you know that they’re not going to hurt themselves and that was my biggest biggest worry with her.” [Caregiver 7]

### Other management strategies

Caregivers reported on the effectiveness of medication, sleep hygiene and adapting the sleeping environment as management strategies for their child’s poor sleep in the online survey (see Table 3). Of these, the most effective strategy appeared to be use of medication. Some caregivers reported receiving help with these strategies from councils, social work, psychology and occupational therapy teams as well as charities such as Newlife and the Smith–Magenis Syndrome (SMS) Foundation UK. However, the majority of families did not and reported planning and paying for adaptations themselves.

**Table 3:** Management strategies implemented by thirty-nine caregivers and their effectiveness reported in the online survey

		Level of improvement reported following implementation of strategy					
	Not implemented	No improvement	Slight improvement	Somewhat significant improvement	Moderate improvement	Extremely significant improvement	Number who received professional input for strategy
Medication	8	7	4	5	5	10	-
Examples listed	Acebutolol, alimemazine, aripiprazole, atenolol, atomoxetine, cannabidiol, chloral hydrate, clonidine hydrochloride, desmopressin, doxycycline, fluoxetine, guanfacine, melatonin, methylphenidate, omeprazole, promethazine, ramipril, risperidone, tasimelteon, trazodone						
Sleep hygiene	12	8	5	7	4	3	12
Examples listed	Same routine, black out blind, earlier bedtime than peers, warm shower later afternoon						
Adapted sleeping environment	17	4	1	6	3	8	9
Examples listed	Stair gates, removed furniture, council funded extension and house renovation, light switch on a timer						
Other	36	0	0	1	0	2	-
Examples listed	Feeding through the night, adapting sleep arrangements to fit with child, holistic treatment						

From the caregiver interviews, three main strategies emerged: adapting sleeping patterns to ‘take turns’ with a partner in caring for the child with SMS overnight, use of medications, and sleep hygiene principles. Two families also reported co-sleeping with their child for at least part of the night, and one family described discouraging daytime napping.

Three families reported that caregivers managed their child’s sleep by taking turns to monitor them, in order to reduce the effects of sleep deprivation on each caregiver.

“So we did sleep in separate bedrooms and sort of swapped over and had a rota that worked for us.” [Caregiver 5]

“So that’s why we have to tag team it, I’ll do the first half, you do the second half, because we both can’t do this together one eye open at the same time, because we’re going to kill each other otherwise. It’s a competition of who’s had more sleep.” [Caregiver 1]

“Yeah so we always had the third bedroom on the go so that the person that was having the night off would have a full unbroken night’s sleep or as much as possible.” [Caregiver 9]

The use of medication to manage sleep was discussed in 6 of the 10 interviews. In three families, the medication was deemed to be helpful:

“Yeah, 1 tablet, 2mg. So she takes that every day and we’ve been doing that for years now, and that seems to regulate things enough for us, which is good.” [Caregiver 3]

In the other three families, medication was discontinued:

“She was on melatonin as a child but not for very long. We didn’t find that it really worked, didn’t really make a lot of difference and I think there was another suggestion that she could have gone on beta blockers but I didn’t bother with that.” [Caregiver 12]

“No, they stopped it up there because they wanted to do tests and it wouldn’t make any difference, I never really thought it did. We had increased it and that made him angry so we went back to slow release which I never liked to stop because in case it made it worse but I pretty much thought that it didn’t make a lot of difference because his sleep patterns never really changed from taking it.” [Caregiver 8]

Similarly, several families reported success with sleep hygiene based management strategies, including ensuring the child had a consistent bedtime routine and sleep-promoting environment:

“I just think a) having a strong bedtime routine is absolutely imperative, b) ensuring that you are clear about it is bedtime. One of the other tips we were given by the sleep consultant was when they’re clinging to you and they don’t want you to go is go out, even if you don’t want to secure them in their room, is just stand outside their room and hold that door handle until they are bored silly and they get back to bed and then they get the picture that you’ve gone out the room and you are not coming back in because it’s sleep time and you can stand outside the room crying your eyes out and they’re crying their eyes out the other side but you’ve got to do it, you have got to do it.” [Caregiver 5]

“Any parent with SMS coming out now, I think educate them now on sleep at a very very early age, educate the parents on a good sleep, you know totally blackout, we’ve got blackout curtains in the bedroom, you know, sort of they need to be educated early on and telling them about you know sleep issues or whatever.” [Caregiver 10]

“And likewise, if she was getting up I wouldn’t go into her room and be lying and cuddling her for ages because again it would just be short, back to bed, I go, I go out of the room because otherwise again maybe it’s just an attention seeking thing, she wants the cuddles, the hugs, so no, that’s not the time for that now. But another parent might actually find that quite difficult whereas I was able to say no, as much as I might want to give you a hug now it’s time to sleep and I need to sleep. You’ve gotta be, what do they say, you’ve gotta be cruel to be kind or whatever.” [Caregiver 12]

However, for the majority of families, sleep hygiene strategies were not effective in managing their child’s sleep, and suggested that a different approach was needed for people with SMS:

“Somebody talked about that at the conference didn’t they, having good sleep hygiene. Well do you know what, [she]’s got amazing sleep hygiene, probably better than any child you’ve ever met, but yet she still won’t stay in bed.” [Caregiver 6]

“Because everyone always talks about sleep hygiene, it’s all about sleep hygiene and I find that really frustrating because like I don't actually know what […] more we can provide.” [Caregiver 7]

“Obviously doesn’t make any difference what you do basically, his Smith–Magenis will override everything.” [Caregiver 8]

“Yeah we had the sleep management specialist and she was no use whatsoever…Well we tried to get across the specific […] difficulties that Smith-Magenis children have with specific sleep issues. She wouldn’t have any of that at all, she wouldn’t treat children differently from someone that was just a behavioural thing which they could control.” [Caregiver 9]

“Yeah I totally agree sleep hygiene is fantastic but when I’ve got two children and one sleeps perfectly and one doesn’t and they’re in the same routine it sets your mind off when you then find out there’s a genetic driver underneath it, sleep hygiene does play a part but it’s not a massive part, his wake up time is governed same as everybody else's by a […] circadian rhythm.” [Caregiver 11]

### Respite

In the online survey, only 5/35 (14%) caregivers reported having access to weekly respite for their child with SMS, 6/35 (17%) monthly and 5/35 (14%) less than once per month. The majority of caregivers (19/35, 54%) stated that they did not have access to any respite, despite 17/35 (49%) stating weekly respite would be their ideal provision.

Many of the caregivers interviewed described regular respite as helpful in managing their child’s sleep. However, the interviews revealed a disparity in provision across different areas of the UK.

“We try and have one weekend night a month, so maybe a Friday or a Saturday night. We don’t do two nights in a row.” [Caregiver 3]

“The beauty of the two nights is you get that whole night of relaxation, you can have a relaxing day you can go to bed relaxed and have a proper sleep and then wake up cope with stuff but when you’re constantly on edge because you’re thinking is the phone going to ring it stops you doing a lot of stuff.” [Caregiver 11]

“I never had any difficulties but I know I’m one of the lucky ones. I know, I hear of the stories and they don’t get any, they’ve never had any respite.” [Caregiver 10]

Many families wanted respite but were unable to access this through social services. Those that were eligible often struggled to find suitable provision in their area:

“We were told that the only way we would be able to get overnight respite now is if we were to declare ourselves unable to look after her at night and essentially put her on the at risk register and that’s something we weren’t prepared to do because it only takes, she head-butts the floor and she’s got a bruise on her head, it only takes one social worker not to understand and she’s no longer living with us is she so we wouldn’t, we didn’t put her on the at risk register.” [Caregiver 6]

“No we’ve been told we’re not eligible for it. I’ve had a bit of a nightmare with social services I will be honest, we’ve had three referrals I think to social services for respite or for some help and they literally dug their heels in and said [she]’s too young.” [Caregiver 7]

“So it’s not about money. There’s nobody out here locally, that is willing to come and sit in our house, go to sleep at night, let us sleep somewhere else, just so they can monitor him once a night, so we can get one night’s sleep a month. There’s nobody out there.” [Caregiver 1]

“I’m in conversation with the social worker to see if we can get direct payments so we can find someone who will actually come and sleep here but even that is finding somebody, you know, direct payments are a great idea but actually where are these people who are queuing up to do these jobs that have the skills to be able to do it.” [Caregiver 5]

## Discussion

This study represents the first detailed investigation of caregiver experiences of managing sleep in people with SMS, a syndrome with significant sleep disturbance and a behavioural phenotype of elevated impulsivity, self-injury, aggression and temper outbursts. The mixed-methods approach using an online survey developed in collaboration with relevant parents and professionals, and a semi-structured interview to gain a broader understanding of caregiver experiences, strengthens the validity of the findings and allows exploration of the complexity of the behavioural phenotype in SMS in relation to sleep management. By describing the cumulative and interactive effect of components of a behavioural phenotype which may underpin the severe sleep management problem in SMS, our findings highlight the need for syndrome-sensitive approaches to caregiver support for sleep management.

Overall, the results clearly indicate that caregivers experience substantial difficulty in managing the sleep of their children with SMS, though some improvements are reported as children get older. Given the complexity of the behavioural phenotype of SMS, these sleep management difficulties are likely influenced by biological, cognitive and social factors which combine to produce unique interactive and summative effects [28]. This syndrome related complexity likely makes poor sleep less amenable to standard intervention approaches which do not take into account the specific difficulties faced by people with SMS and their families. For example, the use of a standard behavioural intervention for insomnia, such as graduated extinction (where caregivers do not attend to the child overnight until an agreed checking time has elapsed; [29], is arguably neither ethical nor feasible in the vast majority of people with SMS given the dangerous aspects of the behavioural phenotype [5–7, 30]. It is therefore likely that parental frustration and stress is exacerbated by receiving standardised advice from professionals which does not consider all aspects of the phenotype of the syndrome and the unique complexity of interactive and summative effects. Therefore, SMS specific sleep safety and management guidance is needed.

Almost all caregivers reported that their child’s poor sleep had a significant impact on the child themselves, as well as the caregiver and wider family. In particular, caregivers noted their children’s poor sleep was associated with daytime fatigue and challenging behaviour. This supports earlier work by Dykens and Smith [20] and Fidler et al. [21] which found sleep disturbance to be a major predictor of daytime challenging behaviour in people with SMS. However, some families did note this association was not always direct, with suggestions on the online survey that challenging behaviour may also be linked to anxiety and anticipation of events. Caregivers also suggested sleep and temper outbursts were linked to poor health including pain from constipation and persistent ear infections. This highlights the need for professionals to assess and rule out pain in people with SMS, particularly before trialling behavioural interventions for poor sleep [31].

Unsurprisingly, managing their child’s poor sleep was very difficult for most families who took part, and the majority of caregivers said they experienced stress and sleep deprivation as much more difficult to manage because of their child’s poor sleep. This supports Heald [26] which suggests mothers of children with SMS have higher than normative perceived stress, depression and anxiety which is linked to their *perception* of their own sleep disturbance rather than their objective sleep parameters. As in Foster et al., [19] some families reported relationship difficulties between family members, with concerns that “*life revolves around SMSer*” reducing caregivers’ capacity to spend time with their partner and other children. Although some families reported siblings did experience sleep disturbance as a result of the poor sleep of the person with SMS, others suggested siblings had adapted and were able to sleep better as a result. These insights into family dynamics and difficulties are important considerations for services looking to support the whole family around a child with SMS.

Caregivers reported a range of practices in managing their child’s sleep, including varying use of medication and sleep hygiene principles, and idiosyncratic strategies such as avoiding napping. In particular, caregivers reported concerns around keeping their child safe at night and having to adapt the environment and their own practices to ensure this, given the profile of adaptive functioning, impulsivity, self-injury, aggression and temper outbursts in SMS [5–7, 11]. Common strategies included taking turns with a partner to monitor their child overnight and limit sleep deprivation, using an enclosed bed system and/or restricting access to other rooms in the house overnight. Some families also used safety gates, locks on doors and windows, video monitors and alarm systems to ensure their child was not able to climb out of the house, access kitchen appliances or harm themselves overnight. Fourteen out of thirty-nine caregivers reported that their child’s safety was a top priority for sleep management, so this is important for professionals to note when supporting families through sleep interventions.

Results from the survey suggest people with SMS are prescribed a wide range of medications to aid sleep, and caregivers reported varying effectiveness. Several people with SMS were reportedly treated with a combination of melatonin and acebutolol to manage night time sleep duration and daytime sleepiness [32]. In the interviews, several caregivers described sleep medications as working well, whilst others reported that they had been discontinued as they did not make a significant improvement to the person’s sleep. This suggests regular review of medications used to manage sleep in SMS may be beneficial and indicates that further input is likely needed beyond medication. Sleep hygiene is one intervention approach which is commonly recommended in the wider intellectual disability literature [33] and can be used alongside or in place of medication to aid sleep. Sleep hygiene practices aim to improve sleep by promoting sleep-onset associations, which help children to settle at sleep onset and then re-settle after waking without a caregiver present. Twenty-seven caregivers reported trialling sleep hygiene strategies, including implementing regular bedtime routines and using blackout blinds, but only twelve had received professional input for this, and only three found the strategies to have made an ‘extremely significant’ improvement to their child’s sleep. This is further supported by Trickett et al. [15] who found very high levels of sleep hygiene compliance even in a group of children with SMS with very poor sleep. In that study there were no significant differences in the sleep hygiene scores of the SMS group compared to the typically developing group, despite children with SMS experiencing over an hour and half less total sleep time on average. In addition, there is limited empirical support for interactions between children with SMS and their caregivers at settling [31] which suggests that caregivers are already implementing sleep hygiene practices and yet these are not associated with improvements to children’s sleep.

Furthermore, although some families in both the online survey and caregiver interviews stated the importance of sleep hygiene in managing their child’s sleep, this approach was deemed less effective than medication. For several families, the underlying biological difference in the circadian rhythm of their child with SMS [16, 17] meant sleep hygiene strategies were insufficient, though professionals working with the family rarely acknowledged this. This suggests a broader understanding of poor sleep in SMS is needed, beyond the standard sleep hygiene interventions recommended for children with intellectual disability, emphasising the potential role of the inverted circadian rhythm in SMS. This is in line with recent recommendations in the UK to treat insomnia in children aged 2–17 years with SMS with paediatric prolonged release melatonin, where sleep hygiene strategies have been insufficient. In the USA, where melatonin is available over-the-counter, formulations and efficacy likely vary and thus melatonin use should be considered on an individual basis [34].

Several caregivers described having regular respite as a useful strategy for managing the poor sleep of the person with SMS. Respite emerged as a theme in the caregiver interviews, with the majority of families stating that respite was (or would be) valuable, but in an appropriate setting. Though some families felt their children would benefit most from being in a residential unit, others preferred respite with foster families or through a personal assistant staying in the family home. However, several caregivers acknowledged the difficulties of finding an appropriate carer who would understand their child’s needs. Several caregivers also described their ineligibility for respite, despite experiencing extreme difficulty in managing their child’s sleep. These findings suggest a ‘postcode lottery’ in UK respite provision and support for families caring for people with SMS which should be addressed. Similar findings are reported more broadly for carers of disabled children in Clements and Aiello’s [35] study of local authority protocols in England. Taken together, these findings suggest a need for new statutory guidance to support disabled children. Caregiver preferences around suitability of the setting and individuals involved in providing respite should also be taken into account when coordinating provision.

Despite the novelty and importance of these findings, there are a number of limitations to the study. Firstly, the interview findings, whilst revealing important caregiver concerns, priorities, strategies and experiences, are limited to parents and siblings caring for people with SMS in a UK context. These findings will usefully inform UK service provision, but may not be as relevant in other contexts, for example where healthcare is not nationally funded, or melatonin is available without prescription. However, the results from the online survey, which formed the basis of the semi-structured interview schedule, were completed internationally and highlight key similarities in experiences across contexts. Secondly, there is a possibility of response bias whereby caregivers who experienced more difficulty in managing their child’s sleep may have been more likely to participate in the survey and interviews. It should also be noted that many participants were recruited through syndrome support groups and conferences, and thus may have greater need for and/or access to support and services than those not involved in these groups. However, given the range of experiences with service provision, safety strategies and management approaches reported, this seems unlikely. Despite this, the survey results likely over-represent the views of caregivers in higher income countries and therefore may not be generalisable to caregivers in other countries who may not have access to the same sleep management strategies (including a separate sleeping space for their child, respite, or medication) or hold the same cultural expectations around solo sleeping. Future research would therefore benefit from including standardised measures of caregiver stress, health and socioeconomic factors to further contextualise the findings. Finally, the survey asked about caregivers’ experience of sleep management across their child’s lifespan and thus findings about change over time are reliant on retrospective memory. The majority of caregivers reported that they were currently caring for an adult with SMS, and thus may be less likely to recall their caregiving experiences during the earlier years of their child’s life as accurately as their current experiences.

## Conclusions

This study is the first investigation of sleep management practices amongst caregivers of people with SMS, a syndrome characterised by a profile of marked sleep disturbance, impulsivity, self-injury, aggression and temper outbursts, preference for caregiver interaction and compromised adaptive functioning. Findings from the online survey highlight similar difficulties in managing the sleep of people with SMS across several continents, with notable impact of poor sleep on caregivers, the wider family and people with SMS themselves. Safety concerns and management strategies were also remarkably consistent, suggesting these concerns emerge as result of the behavioural phenotype of SMS. The findings are strengthened and illustrated by the semi-structured caregiver interviews, which highlight the difficulties of sleep management in the UK context, including barriers to respite provision and concerns around services not understanding the differences needed in sleep management and safety strategies for people with SMS. These concerns and priorities must now be addressed by services and professionals working with people with SMS and their families through the development of SMS specific guidance.

## Supplementary Information

**Additional file 1:** Paper version of Caregiver Survey. Understanding caregiver experiences of sleep management difficulties in individuals with Smith–Magenis syndrome (SMS).

## Data Availability

Research data are not shared due to privacy or ethical restrictions.
