# Supplementary material for: Caregivers’ experience of sleep management in Smith–Magenis syndrome: a mixed-methods study
Source: Orphanet J Rare Dis. 2022 Feb 4;17:35. doi: 10.1186/s13023-021-02159-8 (PMC8815225; doi:10.1186/s13023-021-02159-8)
Supplement: Supplementary file 1 — Additional file 1: Paper version of Caregiver Survey. Understanding caregiver experiences of sleep management difficulties in individuals with Smith–Magenis syndrome (SMS). [file 13023_2021_2159_MOESM1_ESM.pdf]

Understanding caregiver experiences of sleep management difficulties in individuals with Smith-Magenis syndrome (SMS)

1) What is your relationship to the child/person you care for with SMS?

2) How many individuals do you care for (including children, dependants and the child/person you care for with SMS)?

3) Does the child/person you care for with SMS currently live with you?

☐ **Yes** ☐ **No**

If selected yes, please continue to question 3.1. If selected no, please skip to question 4.

3.1) Does the child/person you care for with SMS share a bedroom with any other members of your family (e.g. siblings, caregivers)?

☐ **Yes** ☐ **No**

3.2) How long has this shared sleeping arrangement been in place? Please provide details in the space below.

3.3) Please provide additional details below if there are shared sleeping arrangements outside of the family home (e.g. placement at residential school, shared residence following divorce/separation).

4) How old is the child/person you care for with SMS?

Please select at most one answer

- ☐ Toddler (0-3 years)
- ☐ Young child (4-8 years)
- ☐ Older child (9-12 years)
- ☐ Adolescent (13-17 years)
- ☐ Adult (18+ years)

4.1) In relation to the sleep difficulties listed below, please rate on a scale from 1 (not at all difficult) to 5 (extremely difficult) how difficult each of these items are/have been to manage as a caregiver of an individual with SMS at each given age.

1 = not at all difficult
2 = slightly difficult
3 = somewhat difficult
4 = moderately difficult
5 = extremely difficult

0 = behaviour does not/did not occur

	Settling problems (e.g. refusing to settle/taking a long time to settle)	Sleeping too little during the night	Sleeping too much/often during the day	Daytime sleepiness (e.g. fatigue, lack of energy, drowsiness)	Bedtime resistance (e.g. refusing to go to bed/stay in bedroom)	Abnormal sleep behaviours (e.g. nightmares, night terrors, sleep walking, self-injury)	Waking(s) during the night	Early morning waking	Other*
Toddler (0-3 years)									
Young child (4-8 years)									
Older child (9-12 years)									
Adolescent (13-17 years)									
Adult (18+ years)									

***Other (please state):**

5) How have your experiences of managing sleep difficulties outlined in the previous question changed over time? Please rate using the scale from 1 to 5 outlined below.

1 = A lot less difficult to manage now compared to previous years
2 = A bit less difficult to manage now compared to previous years
3 = No change in management difficulty compared to previous years
4 = A bit more difficult to manage now compared to previous years
5 = A lot more difficult to manage now compared to previous years

0 = behaviour does not/did not occur

Settling problems (e.g. refusing to settle/taking a long time to settle)	Sleeping too little during the night	Sleeping too much/often during the day	Daytime sleepiness (e.g. fatigue, lack of energy, drowsiness)	Bedtime resistance (e.g. refusing to go to bed/stay in bedroom)	Abnormal sleep behaviours (e.g. nightmares, night terrors, sleep walking, self-injury)	Waking(s) during the night	Early morning waking	Other*

***Other (please state):**

6) Have your overall experiences of managing sleep difficulties in the child/person you care for with SMS changed over time? Please provide details in the space below.

7) Has anything consistently improved the quality of sleep in your child/person you care for with SMS? Please provide details in the space below.

8) Has anything consistently reduced the quality of sleep in your child/person you care for with SMS? Please provide details in the space below.

9) Please rate the extent to which sleep difficulties impact your child/person you care for with SMS on a scale from 1 (no impact) to 5 (extremely significant impact).

1 = no impact
2 = slight impact
3 = somewhat significant impact
4 = moderate impact
5 = extremely significant impact

No answer = sleep difficulties do not occur

(●) 1 ( ) 2 ( ) 3 ( ) 4 ( ) 5 ( ) No answer

If selected 1, 2, 3, 4 or 5, please continue to question 9.1.
If selected 'No answer', please skip to question 10.

9.1) Please provide details in the space below of the worst experience(s) your child/person you care for has faced as a result of sleep difficulties associated with SMS.

10) Sleep difficulties may affect your child/person you care for in some ways, as outlined below. Please rate on a scale from 1 (much less difficult) to 5 (much more difficult), the extent to which these items become more/less difficult to manage in the child/person you care for with SMS, as a result of their sleep difficulties.

1 = much less difficult
2 = slightly less difficult
3 = neither more or less difficult
4 = slightly more difficult
5 = much more difficult

0 = item does not occur at all in child/person you care for with SMS

	**Difficulty rating (0-5)**
Challenging daytime behaviours (e.g. self-injury, aggression)	
Relationship difficulties with family members	
Relationship difficulties with peers	
Daytime fatigue	
Problems coping at school/college/daytime placement	
Physical health problems	
Other*	

***Other (please state):**

11) Please rate the extent to which sleep difficulties shown by the child/person you care for with SMS impact you on a scale from 1 (no impact) to 5 (extremely significant impact).

1 = no impact
2 = slight impact
3 = somewhat significant impact
4 = moderate impact
5 = extremely significant impact

No answer = sleep difficulties do not occur

(●) 1 ( ) 2 ( ) 3 ( ) 4 ( ) 5 ( ) No answer

If selected 1, 2, 3, 4 or 5, please continue to question 11.1.
If selected 'No answer', please skip to question 12.

11.1) Please provide details in the space below of the worst experience(s) you have experienced as a caregiver managing sleep difficulties in the child/person you care for with SMS.

12) Please rate on a scale from 1 (much less difficult) to 5 (much more difficult), the extent to which these items are more/less difficult for you as a parent/caregiver to manage, as a result of sleep difficulties shown by the child/person you care for with SMS.

1 = much less difficult
2 = slightly less difficult
3 = neither more or less difficult
4 = slightly more difficult
5 = much more difficult

0 = item does not occur at all

	**Difficulty rating (0-5)**
Stress	
Sleep deprivation	
Relationship difficulties with family members	
Relationship difficulties with friends/colleagues	
Problems completing work/day-to-day tasks	
Physical health problems	
Difficulty concentrating when driving	
Other*	

***Other (please state):**

13) Sleep difficulties can also significantly impact other members of the family, particularly spouses and siblings. Please rate on a scale from 1 (much less difficult) to 5 (much more difficult), the extent to which each of these items are more/less difficult to manage for other members of your family, as a result of the sleep difficulties shown by the child/person you care for with SMS.

(Please leave columns blank if you are a single parent/caregiver and/or the child/person you care for does not have siblings).

1 = much less difficult
2 = slightly less difficult
3 = neither more or less difficult
4 = slightly more difficult
5 = much more difficult

0 = item does not occur at all for spouse/sibling(s)

	**Spouse/partner (0-5)**	**Sibling(s) (0-5)**
Stress		
Sleep deprivation (e.g. through sibling waking)		
Relationship difficulties with others		
Increased behavioural problems (e.g. amongst siblings)		
Problems coping at work or school		
Physical health problems		
Difficulty concentrating on day-to-day tasks (e.g. driving, homework)		
Other*		

***Other (please state):**

14) What do you typically do to keep your child/person you care for with SMS safe at night?

15) Has there ever been a specific situation in which there was a risk to the safety/well-being of the child/person you care for with SMS, as a result of their sleep difficulties? Please provide details/examples in the space below.

15.1) Have these experiences changed/affected the way(s) in which you keep your child/person you care for with SMS safe at night? If so, how?

16) Please indicate on a scale from 1 (no improvement) to 5 (extremely significant improvement) which of the following strategies/interventions/techniques (if any) have improved your experiences of managing sleep difficulties in the child/person you care for with SMS.

1 = no improvement
2 = slight improvement
3 = somewhat significant improvement
4 = moderate improvement
5 = extremely significant improvement

0 = strategy/intervention/technique has not been implemented

	**Improvement rating (score 0-5)**
Medication	
Sleep hygiene strategies	
Adapted sleeping environment	
Other*	

***Other (please state):**

If scored 1-5 for medication, please answer 16.0

If scored 1-5 for sleep hygiene strategies, please answer 16.1

If scored 1-5 for adapted sleep environment, please answer 16.2

If scored 0, please skip to question 17.

16.0) Please list medication use:

16.1) SLEEP HYGIENE STRATEGIES. Please detail below which charities/funding bodies/organisations provided these additional means of support.

16.2) ADAPTED SLEEPING ENVIRONMENT. Please detail below which charities/funding bodies/organisations provided these additional means of support.

17) Please indicate below whether your family has access to overnight respite provision. In the first table indicate current access to overnight respite provision and in the second table indicate the amount of overnight respite provision you would ideally like to have access to.

Please select one answer

	We currently have access to this amount
At least once a week	◯
At least once a month	◯
Less than once a month	◯
None	◯

Please select one answer

	We would like access to this amount
At least once a week	◯
At least once a month	◯
Less than once a month	◯
None	◯

18) Please indicate in the table below whether you have accessed any of the following resources (one response per resource).

Select 'Have received' for any resources you <u>have/had access to.</u>

Select 'Would like to receive' for any resources you <u>do not have access to BUT would like to have access to.</u>

Select 'Neither' for any resources you <u>do not have access to AND would not like to have access to.</u>

	Have received	Would like to receive	Neither
Information about sleep management (e.g. sleep guides, websites)			
Input from health professionals (e.g. GP, paediatrician)			
Behavioural support to child/person you care for from Psychologist			
Access to specialist sleep clinics/courses			
Access to specialist behavioural clinics/courses			
Self-help literature relating to sleep management			
Input from non-sleep professionals (e.g. CAMHS/AMHS)			
Input from sleep specialists (e.g. sleep practitioners)			
Information on how to access sleep services			
Support from other parents/caregivers relating to management of sleep difficulties			
Support in implementing a behavioural intervention			
Prescription of medication to child/person you care for			
Prescription of medication to parent/caregiver			
Professional support relating to own mental health			
Caregiver support via non-profit organisations (e.g. social media groups, family support groups)			
Caregiver support via family/friends			
Caregiver support from health professional/Psychologist			
Other*			

***Other (please state):**

18.1) Based on the resources you selected in the first column (if any), which were most helpful to you and your family?

19) What are your top three priorities for sleep management?

	**Top priorities**
1	
2	
3	

19.1) Any other comments relating to sleep difficulties or safe-sleeping practices in SMS?

20) Are you willing to be contacted by the research team for a follow-up telephone/Skype call (approximately 15 minutes duration) to offer further information about the answers you have provided? This will take place at a time that is convenient for you. There is no obligation to agree to this and the answer you give will not affect current/future research participation with the Cerebra Centre for Neurodevelopmental Disorders.

☐ **Yes** ☐ **No**

If answered yes, please continue to question 20.1

20.1) When would it be best to contact you? Provide details of convenient times and specific dates in the spaces provided.

Check any that apply

**Monday** ○	
**Tuesday** ○	
**Wednesday** ○	
**Thursday** ○	
**Friday** ○	
**Saturday** ○	

You have now completed the survey. Thank you for taking part. If you have any questions about the study or would like any further information, please do not hesitate to contact Stacey Bissell at S.L.Bissell@bham.ac.uk or call 0121 414 9775.

If you have indicated that you are willing to be contacted by the research team to participate in follow-up stages of this study, a member of the research team will schedule these interviews with you using the contact details you have provided.

Please return the completed survey and consent form using the freepost return envelope provided.
